# Exploring contraception myths and misconceptions among young men and women in Kwale County, Kenya

**DOI:** 10.1186/s12889-020-09849-1

**Published:** 2020-11-11

**Authors:** Jefferson Mwaisaka, Lianne Gonsalves, Mary Thiongo, Michael Waithaka, Hellen Sidha, Alfred Agwanda, Carol Mukiira, Peter Gichangi

**Affiliations:** 1 International Centre for Reproductive Health, Mombasa, Kenya; 2 College of Health Sciences, University of Ghana, Accra, Ghana; 3 Department of Reproductive Health and Research including UNDP/UNFPA/UNICEF/WHO/World Bank Special Programme of Research, Development and Research Training in Human Reproduction (HRP), World Health Organization, Geneva, Switzerland; 4 Swiss Tropical and Public Health Institute (Swiss TPH), Basel, Switzerland; 5 University of Basel, Basel, Switzerland; 6 National Council for Populations and Development, Nairobi, Kenya; 7 University of Nairobi, Nairobi, Kenya; 8 African Institute for Development Policy, Nairobi, Kenya; 9 Technical University of Mombasa, Mombasa, Kenya; 10 Ghent University, Ghent, Belgium

**Keywords:** Modern contraceptives, Young men and women, Kenya

## Abstract

**Background:**

Myths and misconceptions around modern contraceptives have been associated with low contraceptive uptake in sub-Saharan Africa and Kenya in particular. Addressing persistent contraceptive knowledge gaps can make a significant contribution towards improved contraceptive uptake among young women. This qualitative study therefore sought to explore and understand young people’s knowledge of modern contraception and to identify their key concerns regarding these methods.

**Methods:**

We used focus group discussions (FGD) with vignette and writing activities to explore key myths and misconceptions around the use of contraceptives. Six FGDs (three for young men and three for young women) were conducted with a total of 28 young women and 30 young men from Kwale County, Kenya. We included 10 discussants aged 18–24 per FGD, one FGD had 8 participants. Predefined codes reflecting the discussion guides and emerging issues in the FGDs were used to develop the thematic coding framework. Our analysis followed a pattern of association on the key preset themes focusing on myths and misconceptions around contraceptive use.

**Results:**

Results are presented under four key themes: awareness of contraception, myths and misconceptions around contraception, males’ contraceptive narratives and young people’s preferred sources of contraceptives. Both men and women participants reported basic awareness of contraceptives. A mixture of biological and social misconceptions were discussed and included perceptions that modern contraception: jeopardized future fertility, could result in problems conceiving or birth defects, made women promiscuous, was ‘un-African’, and would deny couples their sexual freedom. Compared to female respondents in the study, young men appeared to be strong believers of the perceived socio-cultural effects of contraceptives. On preferred sources of contraceptives, respondents reported on two main sources, pharmacies and public hospitals, however, they could not agree on which one was suitable for them.

**Conclusions:**

This study revealed the presence of a mixture of biological and social myths and misconceptions around contraception, with young men also strongly adhering to these misconceptions. The low level of contraceptive knowledge, particularly on contraceptive fears as revealed by the study demonstrate critical gaps in sexual and reproductive health (SRH) knowledge among young people. Improved SRH literacy to address contraceptives’ fears through appropriate and gender specific interventions to reach out to young men and women with factual SRH information may therefore contribute to increased uptake of SRH services including modern contraceptive methods.

**Supplementary Information:**

The online version contains supplementary material available at 10.1186/s12889-020-09849-1.

## Background

Unmet need for modern contraceptives in sub-Saharan Africa (SSA) is high, with an estimated 53 million (60%) of 89 million women in need of contraceptives [1]. This need for contraception is often higher among young women. In Kenya, in particular, young people below the age of 25 constitute approximately 66% of the country’s population, and often experience some of the poorest sexual and reproductive health (SRH) outcomes [2]. The Kenya Demographic and Health Survey (KDHS) of 2014 indicated that 18% of adolescent girls aged 15–19 were already mothers or were pregnant with their first child [3]. Childbearing was reported to begin early in Kenya with almost one quarter of women giving birth by age 18 and approximately half by age 20 [3]. Contraceptive prevalence rate among sexually active unmarried girls aged 15–19 years in Kenya is 49 and 64% among those aged 20–24 years [2, 3]. Moreover, the recent KDHS established that 12% of young women and 21% of young men aged between 15 and 24 years had their sexual debut before age 15, while 47% of young women and 55% of young men between the ages of 18–24 years had their first sexual intercourse before their 18th birthday [3]. The low prevalence rates of modern contraceptives coupled with early sexual debut increases the potential for unintended pregnancies among young women.

Young women in SSA and Kenya, in particular, are often deterred from using modern contraceptives because of: limited access to contraceptive services; poor quality of available services; limited choice of methods; fear of side-effects; cultural or religious disapprovals; users’ or providers’ biases; and gender-based barriers [4, 5]. Additionally, myths and misconceptions about the suitability of certain methods to particular audiences, such as views by individuals and some healthcare providers that long-acting methods are unsuitable for younger women, contribute to non-use, discontinuation and low uptake of these methods by young women [6]. Increasing the level of contraceptive use, especially the use of long-acting reversible methods, can have a significant impact on unplanned pregnancies largely affecting young women [7]. Moreover, addressing contraceptive knowledge gaps and misperceptions can contribute towards improved contraceptive uptake among young women [4]. A multivariate analysis examining associations between modern contraceptive uptake and belief in myths for individuals and communities in Kenya, Nigeria and Senegal found a negative association between belief in myths and contraceptive use in all the three countries. In Kenya, the study found that, a one-point increase out of a total 8 points in the number of myths believed by a woman was associated with a 35% decrease in her likelihood of using any modern method. Furthermore, Kenya was found to have the highest proportion of men and women agreeing with family planning myths [8]. Similar concerns around long-term fertility impairment and dangers of prolonged use have been observed in both urban and rural Kenya [9]. Comparably, a cross-sectional study in Ethiopia determined that women who had no myths and misconceptions towards long acting and permanent contraceptives (LAPMs) were more likely to use LAPMs compared to their counterparts who believed in contraceptive myths and misconceptions [10]. Most personal experiences of contraceptive use in Kenya include IUD, injectables pills, implants and male condoms [4]. Injectables however are the most widely used followed by implants and the pill across all sexually active women of different age groups and other demographic characteristics including marital status [3].

Despite prior research on contraceptive uptake associating male partner’s knowledge and attitudes towards modern contraceptives with women’s contraceptive use [11], most of the studies in Kenya focus on women’s perspectives [12]. Few studies have captured the contraceptive narratives from young men and women in SSA and Kenya in particular. Moreover, young women between the age of 15–24 face a significantly higher burden of unmet need for contraception compared to older women [13, 14] with little insights regarding their contraception perspectives. Therefore, this qualitative study sought to explore and understand both young men’s and women’s knowledge of modern contraception and to identify key concerns regarding the use of contraceptives. Findings from this study will provide additional insights to policy makers and program managers in the design of appropriate age and gender specific SRH interventions aimed at addressing unfounded contraceptive fears and misconceptions.

## Materials and methods

### Study design and setting

This is a qualitative study involving young people (men and women) aged 18–24 conducted in a peri-urban region of Ukunda in Kwale County, Kenya utilizing focus group discussion. These FGDs were part of a larger study [15] which used a randomized controlled trial to assess the effect of a mobile health intervention that provided sexual and reproductive health information to young people via text messages on their mobile phones in Kenya and Peru. The FGDs took place prior to the RCT and aimed to confirm key myths and misconceptions around contraceptive use and common reasons for not using them.

The study’s formative phase [16] included young people between the ages of 15–24, but established that a majority of young people aged 15–17 years did not own their own phones (instead shared them with siblings and other relatives). Mobile phone ownership was an established eligibility criterion for this mobile health intervention study; therefore, for subsequent stages (including these FGDs), the decision was made to limit participation to those between 18 and 24.

### Study procedures

Purposive sampling was used to select study participants residing in the study area. Resident enumerators in the larger digital health study were used to recruit study participants. Participants were identified based on their specific characteristics including participants’ ability to communicate their sexual life experiences in an articulate manner, relates to the culture of the targeted community and their willingness to participate. Eligibility was based on age ranging 18–24 years for both sexes, literacy levels (able to read and write) and being a resident of Kwale County. The ability to read and write regardless of any schooling level was considered for these FGDs since participants were required to anonymously provide written responses prior to starting the group’s discussions. The individual responses explored independent thoughts from the participants which guided the discussions. Three interviewers, (note taker, moderator and one doing the additional writing activities) were trained to facilitate the discussions and FGD activities; they were provided with semi-structured interview guides.

We conducted six FGDs (three for young men, three for young women) with 28 young women and 30 young men from Kwale County. We included an average of 10 discussants aged 18–24 per FGD. The discussions were held at the community social halls in Kwale County and were facilitated by moderators of the same sex. Our FGDs were dynamic, with vignette and private writing activities to explore individual’s and groups’ key myths and misconceptions around the use of contraceptives. Young people were asked to share what they knew about contraception with the following prompt:

### Tell me what “contraceptive” means or what you understand by “contraceptive”

An additional activity asked participants to react first individually, and then as a group, to the following vignette:

***Vignette:*** *Omar and his partner Mwanakombo are talking about using family planning, but they are nervous about what they have heard from friends. What are some of the things they may have heard which could make them nervous*?

After the moderator read the above vignette, participants wrote their responses on large cards – an anonymous, individual activity. A second data collector gathered participants’ contributions and laid out the responses (grouping similar contributions) for the participants’ review. Each FGD group was then asked to discuss and select the most common concerns that young people in their community agreed with.

A semi-structured discussion guide (additional file: Tool 1 Focus Group Discussion Guide for Youth 18–24) developed specifically for this study was used, allowing the phrasing and focus of the questions on each topic and method mentioned by participants to be tailored depending on the group. In addition to the vignette discussions, young people in the study were asked to name and describe the kind of contraceptives they’ve heard of and where people their age could get contraceptives. Additional discussions involved qualities of an ideal contraceptive-dispensing unit specifically in non-clinical settings as well as what young people thought could be done to improve their contraceptive uptake. All discussions were held in Swahili, and all proceedings were audio recorded, with the participants’ consent.

### Data management and analysis

The discussions were transcribed verbatim, translated into English, coded and analyzed thematically using NVivo version 12. Two researchers (JM and CM) read through all the six transcripts independently and held a session to discuss and agree on overarching themes. A set of predefined codes reflecting the discussion guide as well as emerging issues was used in coding the qualitative data. This was followed by a thematic coding framework in assessing the transcripts. Our analysis followed a pattern of association on the key preset themes focusing on awareness, and myths and misperceptions around contraceptives.

Awareness referred to one’s ability to identify (name) contraceptives without necessarily understanding how the contraceptive works. Myths in this study refer to misinformation and widely held but false beliefs about contraception that differ from women’s concerns regarding documented or experienced method-related side effects. Finally, misconceptions are defined as specific and widespread beliefs about the effects or purpose of contraceptives that are not supported by any scientific evidence [6]. Our analysis and findings are presented in accordance with the Consolidated Criteria for Reporting Qualitative Research (COREQ) [17].

### Ethical considerations

Ethical clearance was obtained from World Health Organization Institutional Review Board (Protocol WHO A65892 core) and the Kenyatta National Hospital-University of Nairobi Ethics and Review Committee (KNH ERC P550/09/2014). All participants gave written informed consent to participate in the study.

## Results

Among youth participants (Table 1), 56.7% of male respondents reported to have completed their secondary education compared to 28.6% of females. When disaggregated by age and sex groupings, the proportion of females in dating relationships was relatively high compared to their male counterparts (46.4 and 26.7%, respectively). In this study, those dating or reported as friends with benefits referred to a participant who had a regular sexual partner with whom they enjoyed spending time, without any formal agreement to marry. Married or engaged, on the other hand was reported by those with sexual partners and in unions with formal marital arrangements. There were more Muslim female participants (82.1%) than Christians (17.9); by contrast, all male participants were Muslims. This study was done in Kwale County where the Digo community who are predominantly Muslims are the majority. This explains the higher proportion of Muslim participants in the study.

**Table 1:** Sociodemographic characteristics of study participants, by sex (*N* = 58)

	Youth Males n (%)	Youth Females n (%)
**Age range in years**	All (18–24) 30 (100%)	All (18–24) 28 (100%)
**Highest school level**		
Primary incomplete	1 (3.3)	2 (7.1)
Primary complete	1 (3.3)	7 (25.0)
Secondary incomplete	5 (16.7)	3 (10.7)
Secondary complete	17 (56.7)	8 (28.6)
Tertiary/college	6 (20.0)	8 (28.6)
**Relationship status**		
Single	20 (66.7)	13 (46.4)
Dating/Friends with benefits	8 (26.7)	13 (46.4)
Married/Engaged	2 (6.6)	2 (7.2)
**Religion**		
Muslim	30 (100)	23 (82.1)
Christian		5 (17.9)

Results are presented under four key themes: (i) awareness of contraception; (ii) myths and misconceptions around contraception; (iii) males’ contraceptive narratives and young people’s preferred sources of contraceptives.

### Awareness of contraception

When asked to explain what they understood by contraceptives, both males and females reported awareness of contraceptives, with some providing a combination of descriptions and/or listing of the methods. Injections and the intrauterine device (IUD or the ‘coil’, as it was called by respondents in the study) were the most frequently identified contraceptives. Other methods mentioned included condoms, withdrawal and rhythm method of birth control. It was clear that young people knew or had heard about contraceptive methods, but had minimal knowledge on how they actually worked.

*There are injections that you can use, I don’t know how to explain it to you. There are injections when one uses, it will just help you to family plan* (Respondent 4, young women FGD 001)

*There is something I have heard but I’m not familiar … with a coil, when you get to a hospital, you get the coil … they put coil to women* (Respondent 9, young men, FGD 003)

Compared to young men, young women were more aware of contraceptives in that, not only did they list the contraceptives but also went ahead to explain the perceived duration of effectiveness of some methods like the injection and implant.

*To add more on the injection, there is an injection that someone can have for 3 months and there is the implant … which is put here (shows her arm) for 5 years* (Respondent 6, young women, FGD 001)

### Myths and misconceptions around contraception

Male and female participants shared several myths and misconceptions around contraception. Some respondents mentioned that the use of contraceptives jeopardized future fertility and could lead to serious health complications such as prolonged menstrual bleeding, problems conceiving, and birth defects. The most common misconception among both male and female participants was the perceived infertilities mistakenly associated with contraceptives. Hearsays from peers about perceived side effects and misinformation came out strongly in both male and female FGDs. This finding might explain albeit tacitly young people’s source of contraceptives’ myths and misconceptions.

*People say that when you get the injection and if it does not work well for you, you bleed. You will bleed until you cannot get pregnant again and give birth. You will just be bleeding and bleeding, there are people who bleed for many months because of those injections.* (Respondent 1, young women, FGD 001)

*If for example you want to use the-after-three-months injection they say that if you use it often, then time comes and you want to stop using it and you want to get pregnant, you may wait for ten good years or forever and you will not get a baby at all. Because … I don’t know it makes the egg to get lost and it becomes weak that is what it means by destroying the womb*. (Respondent 6, young women, FGD 001)

*When they have used contraceptive to prevent them from getting pregnant, if a man and a woman, maybe in some years to come they will have stopped using them and they now want to have children, some of them (children) will be born with abnormalities, not as usual children but deformed and underweight.* (Respondent 9, young men, FGD 001)

Participants also reported fears that IUDs could be pushed inward during sexual intercourse and damage the women’s reproductive organs. This came from the belief that the perceived ‘discomfort’ during sexual intercourse inaccurately believed to be experienced by users of IUDs is as a result of the IUD moving out of its normal position thereby making it ‘exposed’ during sexual intercourse.

*I heard about the coil, that coil is inserted here in the womb, the time you are having sex with that person and he pushes it inside already he would have messed up everything, it will force you to remove it.* (Respondent 4, young women, FGD 001)

### Men’s contraceptives narratives

Men in the study had their own strong concerns about adverse socio-cultural effects of contraceptives. Several of these related to sexual relations between couples and sexual desire. Some reported that contraceptives contribute to decreased sexual desires among women, ‘forcing’ men into infidelity.

*Other negative effect is that, it breaks marriages because those drugs lower women’s sexual feelings, so if you (as a man) were used to like four sex rounds a week, this will reduce to two times, it will be a must for you to go outside you will not agree.* (Respondent 4, young men, FGD 002)

Somewhat counterintuitively, contraceptives were also perceived to contribute to infidelity on the part of *women*. As a result, male respondents worried about the effect of contraception on the trust in a relationship. This was seemingly because, with reduced risks of getting pregnant, young men thought that their women will increase their sexual frequency and with more partners. This goes further to explain the contraception confusion displayed by young men; while others opined that contraceptives would decline their sexual rights due to their women’s reduced sexual desires, their counterparts in the same age group had contrasting beliefs relating to the sexual effects of contraceptives.

*We have trusted one another, and the wife takes those contraceptives and prevented herself from pregnancy, there will be no trust between us because one (wife) knows that she can have sex with anyone from outside and not getting pregnant, so I will not trust her* (Respondent 2, young men, FGD 001)

*In short it means untrustworthiness because you cannot get pregnant, … so maybe you will be having sex with someone or feel free to have sex anyhow and thereby infecting your partner with sexual diseases* (Respondent 2, young men, FGD 002).

Interestingly, male participants also perceived that contraceptive methods deny couples their sexual freedom and regarded them as an unnecessary burden. Respondents were concerned about the implied prerequisite of always attaching contraception to sex, perceiving their sexual lives to be ‘enslaved’ to contraception thereby taking away the pleasure of having sex.

*I see it as slavery using them, because it will be you and your wife at home and the time you do the marriage act (sex) you will be wearing trust (condom) and then using drugs every time you cannot skip, if you skip it will be a problem, when you say that you are leaving them (family planning pills) also it is a problem. Again, every time you will be going to the hospital or going to the chemist and take drugs, as in a burden, something like that.* (Respondent 4, young men, FGD 001)

Among additional socio-cultural concerns, young men in the study also expressed beliefs that it was against ‘African’ traditions to NOT want children.

*First of all, in Africa, Many Africans perceive contraceptives as un-African … In the African communities, children are important … if someone avoids getting a child …, the first year no child, second year no child … husband will now start to worry … And due to that the woman will be divorced* (Respondent 6, young men FGD 003)

Finally, young men confused abortion and pregnancy prevention methods, with some participants mentioning that contraceptives could also be used to terminate pregnancies. Some young men in the study reported that by preventing “natural pregnancy progression” after unprotected sex, emergency contraceptive pills served to end an ‘existing pregnancy’. It was also established that some participants in the men FGDs couldn’t tell the difference between emergency contraceptive pills that prevent pregnancy after unprotected sex and abortion pills that terminate an already existing pregnancy.

*Contraceptives are things which prevent one from getting pregnant or if one wants to abort when she has been impregnated by a man* (Respondent 2, young men FGD 001)

This confusion resulted in some participants feeling contraceptives were a ‘curse’.

*You can say it is a curse because it is like doing murder, you will have killed, you can get a curse from God because you are not allowed to kill another for any mistake* (Respondent 3, young men, FGD 003)

### Young people’s preferred sources of contraceptives

Although pharmacies and public health facilities were the reported common sources of contraceptives among the respondents, there was no consensus as to where young people would prefer to go for contraceptives. Both male and female respondents expressed varied preferences and dissatisfactions with the two contraceptive sources. These variations ranged from lack of privacy at the public hospitals, attitude of the healthcare providers to the cost associated with getting their preferred methods. Some respondents preferred pharmacies (locally referred to as “chemists”) to public facilities because of their privacy nature.

*The goodness of the chemist is that there is privacy, because everyone who sells at the chemist is not known to you, but you can go to the hospital and you happen to find relatives who might see you, now when you go to the chemist, one is not asked what they are going to buy* (Respondent 3, young women, FGD 001)

Being served by people their age was a reported factor that made pharmacies appeal to young people. Moreover, respondents reported that pharmacies are the preferred sources of contraception because the pharmacies’ personnel were considered polite and welcoming as opposed to the reported attitudes displayed by healthcare providers at the public health centres.

*Then another reason is that most of those attendants at the chemist are age mates. So when you get there you can express yourself in details and he/she will understand you, unlike at a hospital where you will find an old person is the one serving people.* (Respondent 10, Young women, FGD 003)

*As my colleague had said, you can get a harsh person at the hospital but when getting to the chemist most of them talk politely and welcoming the customers, they have good language* (Respondent 7, young women, FGD 002)

Conversely, and unlike in the previous themes where participants acknowledged and almost approved their colleagues’ assertions about contraceptives, the preferred sources presented dissenting opinions. Public hospitals were instead considered ideal by a section of the respondents who opined that unlike pharmacies, public hospitals provided better and professional services and at an affordable cost.

*Also at the hospital the services are cheap as compared to places like chemists. You see the hospital might be a government hospital so what he/she needs might be cheap compared to other centers* (Respondent 1, young men, FGD 002)

*There is better treatment at the hospital compared to these chemists, for example if the service provider is a form four leaver (basic O-level graduate) he will not understand anything, but at the hospital they are good and educated nurses and doctors* (Respondent 7, young men, FGD 003)

## Discussions

Most respondents (both men and women) in the FGDs were aware of contraceptives. Nonetheless, findings from this study confirmed that awareness does not translate to accurate knowledge of contraception and how it works. We also identified key myths and misconception that young men and women have against modern methods of contraception. Additionally, we described specific socio-cultural perceptions exhibited by men respondents in the study, which could influence their and their partners’ acceptance and use of contraceptives. Finally, we looked at preferences put forth by young people when choosing where to seek contraceptives’ services.

Uncertainties about side effects play a key role in young people’s disapproval of the effectiveness of modern contraceptives in Kenya [4]. Unsubstantiated fears about contraceptive safety can lead women to either forgo contraceptive use, opt for less effective methods or even use effective methods incorrectly hence further jeopardizing their overall health [6]. Consistent to these findings is a qualitative study on barriers to modern contraceptive uptake among young women in Nyanza, Coast and Central regions of Kenya which established that myths and misconceptions were a major barrier to contraceptive uptake and that individual’s beliefs about the effectiveness of contraceptives were dependent on social approval by their peers [4]. The negative perceived effects of modern contraceptives will ultimately contribute to the low uptake if information about modern contraceptive methods continue to be misrepresented [18].

Misconceptions around future fertility later in life seem to create fear in both young men and women. Prevalence of such myths and misconceptions have also been demonstrated to be existing among young men and women in other studies and national surveys [3, 6, 11, 18–20]. These studies and national surveys established that the perceived interference with future fertility was the main barrier to modern contraceptive uptake among young women. Determining the actual magnitude effect of these myths and misperceptions remains a daunting task, as most quantitative studies fail to distinguish women’s concern about documented side effects from unsubstantiated fears about adverse health effects [6].

Few studies have also attempted to describe the factors contributing to support of modern contraceptive use among sexually active men [12]. In this study young men perceived contraception use to have the potential to create conflicts within marriages and a possible spike in sexually transmitted infections. This was tied to a perception that because women no longer need to worry about pregnancy - the only perceived evidence of a sexual act for women – they would be inclined towards ‘promiscuity’. In Nigeria, it was found that, men’s perceptions that contraceptives are enablers for promiscuous behaviors among women was negatively associated with the use of modern contraception among their female partners [21]. Men’s lack of trust towards their female partners has been shown to be a major driver of misconceptions associating contraceptives with promiscuity [4, 21].

The fact that these concerns were held by male participants underscores the importance of engaging men in addressing low contraceptive uptake. In Kenya, male partners’ opposition was identified as one of the key factors contributing to contraceptives discontinuation tendencies [5]. Similarly, in an analysis of 2005–2009 Demographic Health Survey data from 21 African countries, it was established that gender norms and gender inequalities in the community were among the potential contributors to uptake or non-use of contraceptives [18, 22]. Men therefore need to be adequately involved and informed about contraceptives and their effects, as they remain important decision makers in contraceptive use [12].

As such, a critical strategy to reduce misinformation and increase uptake of contraception among young women is to increase avenues where young women *and young men* can get credible SRH information. Both national and county governments in Kenya and other SSA countries need to provide technical support and supervise the implementation of youth-friendly service interventions in public health facilities. For SRH services to be effective to young people, there is need for high quality reproductive health products, information and services, increased points of accessing SRH services for young people as well as provision of continuous health education, including comprehensive sexuality education, to help them win the war against societal taboos associated with SRH seeking behaviours [13, 18].

Other figures in young people’s lives can also play an important role in normalizing their use of contraception. This study has established that, a section of young people perceived pharmacies’ personnel as better placed to offer friendly contraceptive services compared to the public health facilities. With improved regulations and control of pharmacy services in Kenya, provision of contraception through pharmacies can help address significant barriers associated with young people’s contraceptives uptake [23]. There is also evidence, for example that parent-youth communication on SRH is associated with young people’s improved use of contraceptives [24]. Interventions which include parents and promote increased discussion within families therefore provide another interesting opportunity, with the potential to see significant positive influence on young people’s sexual attitudes, value and beliefs regarding modern contraceptives [25, 26]. In general, there is a need to push and advocate for the design of multilevel programs and interventions targeting young people, particularly young men with SRH information. For young men in particular, this entails going also to where they already gather outside the home, including: youth camps in churches and mosques, or popular media programs targeting young men in Kenya like ‘Man Enough’ and ‘Man Talk’. Using these as information dissemination channels, can contribute to helping young men to understand their SRH roles and be well informed for them to act both individually and collectively to empower their female partners while improving their own SRH behavior.

Moreover, SRH communication programmes should focus on changing contraceptive norms among adolescent boys and girls before misconceptions set in [6]. In addition to early comprehensive sexuality education, one solution to reach young men and women, as determined by several studies, is via direct client communication using a digital health interventions [27–31]. The focus for effective media should be on providing correct information as opposed to negating the myth while keeping the information simple with a compelling explanation to replace the existing myths and misconceptions [6].

### Study strengths and limitations

One significant strength of this study is in the way the FGDs were conducted where in-depth thoughts were first explored independently through vignettes using an anonymous individual activity. This individual reaction by study participants prior to the group discussions allowed each group to focus on issues they considered the most common in their community. This approach provided an expansive method in the analysis of views and feelings considered relevant in this study.

This study had certain limitations. Young people may have felt uncomfortable sharing their views on contraception, particularly if they were not confident in their knowledge. Moreover, they may have been uncomfortable or embarrassed to share myths and misconceptions, particularly if they felt unsure as to whether these were shared. We attempted to mitigate these by providing opportunities for participants to make individual contributions to the discussion. In addition, the use of vignettes allowed participants to place any myths/misconception on a fictional third party. Another limitation might be the eligibility criteria limiting participants by literacy levels, however, according to the KDHS of 2014 [3], literacy rates among 15–24 year olds in Kwale was more than 95%, a significant majority of young people in Kwale County can therefore read and write.

## Conclusion

This study contributes to the larger literature on young people’s myths and misconceptions preventing them from using modern contraceptives. Overall, these findings are consistent with other studies in SSA which imply that improved SRH literacy must include explicit attention to lingering contraception myths and misconceptions. Additionally, efforts to address SRH myths and misperceptions need to employ multifaceted approaches at both individual and community levels. Finally, gender-specific interventions to specifically reach out to young men with factual SRH information aimed at dispelling contraception misconceptions may go a long way towards making young men educated about and supportive of their partners’ choice to use contraception.

## Supplementary Information

**Additional file 1.** Tool 1 Focus Group Discussion Guide for Youth [18–24]

## Data Availability

The datasets used and analyzed in this study are available from the corresponding author upon request.

## Abbreviations

ARMADILLO: Adolescent/Youth Reproductive Mobile Access and Delivery Initiatives for Love and Life Outcomes
FGDs: Focus Group Discussions
KDHS: Kenya Demographic and Health Survey
SRH: Sexual and Reproductive Health
SSA: Sub Saharan Africa
