# Supplementary material for: Exploring contraception myths and misconceptions among young men and women in Kwale County, Kenya
Source: BMC Public Health. 2020 Nov 11;20:1694. doi: 10.1186/s12889-020-09849-1 (PMC7661170; doi:10.1186/s12889-020-09849-1)
Supplement: Supplementary file 1 — Additional file 1. Tool 1 Focus Group Discussion Guide for Youth [18–24] [file 12889_2020_9849_MOESM1_ESM.docx]

ARMADILLO Study

**Study Instrument 1:** Focus Group Discussions

Participants: Youth (18-24)

**Tool 1 Focus Group Discussion Guide for Youth (18-24)**

Researchers will ask participants a series of questions addressing the following topics: myths around contraception, from where young people get family planning, and qualities of ideal FP commodity dispensing in non-service facilities.

The number of questions on each topic, as well as the phrasing and specific focus of the questions will depend on the focus group. This will allow researchers to gather the best input from FGDs by tailoring questions to the background and experience of participants.

Participants will be asked to discuss questions on each topic depending on their background and the need to clarify or follow-up on their responses. Researchers will not ask any questions addressing domains other than those identified here. Below is a list of the domains to be explored in the focus group discussion, along with example questions and probes that may be used to explore each domain.

**Myths around contraception**

Tell me what “contraceptive” means or what you understand by “contraceptive”

**Vignette:** Omar and his partner Mwanakombo are talking about using family planning, but they are nervous about what they have heard from friends. What are some of the things they may have heard which could make them nervous?

***Ranking:*** *Write all given reasons down on large cards, and combine this list with any additional reasons which have been identified (from literature review or national-level documents), ask the group to select the 10 most relevant*

**Where young people get family planning**

- Tell me about all the places in Kwale, where someone can get family planning?
  - Probe: Places that a young person like you could go if they needed family planning (like condoms or other contraception)? *(Write out list)*

*For each listed contraception source:*

- When would a young person choose to go to a ________ to get family planning? *(Probe: after unprotected sex, as a one-off, desiring a long-term contraception etc.)*
  - For what reason would they go?
- Tell me about the kind of young person who would go to a _______ if he/she needed family planning? *(Draw stick figure under each source name, probe on and label with identifiers: gender, marital status, etc.)*
  - Explore gender, type of contraception
- What does this young person like about ______ as a resource for family planning?
  - What are the most important qualities about ______ as a resource for family planning?
- What does this young person dislike about ________ as a resource for family planning?

**Qualities of ideal FP-dispensing in *non-service sources***

*For young people (probe in union or single separately) seeking contraception:*

- What are the most important things a young person *needs* when accessing family planning from a pharmacy or a shop?
- What are the most important qualities a pharmacy or a shop needs to have for a young person to be comfortable obtaining family planning?
  - What could be done to increase the comfort of young people who might not be comfortable going to these establishments?
  - *Ranking exercise to establish top (re: most important qualities of services)*
- What does someone needing contraception from a pharmacy or a shop also want at the point they are seeking access?
- To what extent do they want these add-ons, if they come at the expense of qualities that they value
  - Example: *We’ve talked to other young people who have good suggestions for improving things at pharmacies and shops. They also value things like privacy and speed. Other group have been trying to deal with this – what would you do to make sure young people could get what they needed from pharmacies and shops without being uncomfortable?*
